# Anomaly Detection Based on Sensor Data in Petroleum Industry Applications

**DOI:** 10.3390/s150202774

**Published:** 2015-01-27

**Authors:** Luis Martí, Nayat Sanchez-Pi, José Manuel Molina, Ana Cristina Bicharra Garcia

**Affiliations:** 1 Department of Electrical Engineering, Pontifícia Universidade Católica do Rio de Janeiro, Rio de Janeiro 22451-900, Brazil; 2 Instituto de Lógica, Filosofia e Teoria da Ciéncia (ILTC), Niterói 24020-042, Brazil; E-Mail: nayat@iltc.br; 3 Department of Informatics, Universidad Carlos III de Madrid, Colmenarejo, Madrid 28270, Spain; E-Mail: molina@ia.uc3m.es; 4 ADDLabs, Universidade Federal Fluminense, Niterói 24210-340, Brazil; E-Mail: bicharra@ic.uff.br

**Keywords:** anomaly detection, big data, time-series segmentation, outlier detection, oil industry applications

## Abstract

Anomaly detection is the problem of finding patterns in data that do not conform to an a priori expected behavior. This is related to the problem in which some samples are distant, in terms of a given metric, from the rest of the dataset, where these anomalous samples are indicated as outliers. Anomaly detection has recently attracted the attention of the research community, because of its relevance in real-world applications, like intrusion detection, fraud detection, fault detection and system health monitoring, among many others. Anomalies themselves can have a positive or negative nature, depending on their context and interpretation. However, in either case, it is important for decision makers to be able to detect them in order to take appropriate actions. The petroleum industry is one of the application contexts where these problems are present. The correct detection of such types of unusual information empowers the decision maker with the capacity to act on the system in order to correctly avoid, correct or react to the situations associated with them. In that application context, heavy extraction machines for pumping and generation operations, like turbomachines, are intensively monitored by hundreds of sensors each that send measurements with a high frequency for damage prevention. In this paper, we propose a combination of yet another segmentation algorithm (YASA), a novel fast and high quality segmentation algorithm, with a one-class support vector machine approach for efficient anomaly detection in turbomachines. The proposal is meant for dealing with the aforementioned task and to cope with the lack of labeled training data. As a result, we perform a series of empirical studies comparing our approach to other methods applied to benchmark problems and a real-life application related to oil platform turbomachinery anomaly detection.

## 1. Introduction

The petroleum industry has evolved into a highly-supervised industry, where operational security and safety are present and foundational values. In the particular context of modern offshore oil platforms, almost all of the installed equipment include sensors for monitoring their behavior and remote-controlled actuators to act upon them in order to regulate the operational profile, to avoid undesired events and to prevent possible catastrophic failures. Oil plant automation physically protects plant integrity. However, it acts by reacting to anomalous conditions. Extracting information in an on-line fashion from raw data generated by the sensors is not a simple task when turbomachinery—a key class of equipment—is involved.

The term, turbomachine, applies to any device that extracts energy or imports energy from a continuously-moving stream of fluid, either liquid or gas [1]. Elaborating further, a turbomachine is a power or head generating machine, which employs the dynamic action of a rotating element, the rotor. The action of the rotor changes the energy level of the continuously-flowing fluid through the machine. Turbines, compressors and fans are all members of this family of machines. In contrast to positive displacement machines, especially of the reciprocating type, which are low-speed machines based on the mechanical and volumetric efficiency considerations, the majority of turbomachines run at comparatively higher speeds without any mechanical problems and with volumetric efficiency close to the ideal (100%). This application context calls for the application of anomaly detection methods [2] that grant and supervise the effective and safe usage of the machinery involved.

Anomalies themselves can have a positive or negative nature, depending on their context and interpretation. The importance of anomaly detection is a consequence of the fact that anomalies in data translate to significant actionable information in a wide variety of application domains. The correct detection of such types of unusual information empowers the decision maker with the capacity to act on the system in order to adequately react, avoid or correct the situations associated with them.

Anomaly detection has seen extensive study and use in a wide variety of applications, such as fraud and intrusion detection [3], fault detection in safety critical systems [4], finance [5] or industrial systems [6], among many others (see [2] for a survey).

In the case of industrial anomaly detection, units suffer damage due to continuous intensive use. Those damages need to be detected as early as possible to prevent further escalation and losses. Data in this domain are referred to as sensor data, because these are recorded using different sensors and collected for analysis. Hence, it can be said that, in this context, anomaly detection techniques monitor the performance of industrial components, such as motors, turbines, oil flow in pipelines or other mechanical components, and detect defects that might occur due to wear and tear or other unexpected circumstances. Data in this domain have a temporal aspect, and time series analysis is also used in some works, like [7].

The problem debated in this paper was prompted by the complexity and requirements of the task of the early detection of behaviors that could potentially lead to turbomachine or platform failures in the application context of interest. One additional characteristic of this problem is that these machines have different operational profiles. For example, they are used at different intensities or throttle depending on the active platform operational profile. Therefore, in order to correctly detect future anomalies, it is essential to segment the dataset available in order to automatically discover the operational regime of the machine in the recent past. This segmentation algorithm would allow one to discriminate the changes of the operational profile from anomalies and faults, as manual changes are not logged, and sometimes, those modifications take place without human supervision.

Furthermore, in this particular case, it can be argued that we are also facing a Big Data problem [8]. Each machine has from 150 to 300 sensors that submit information to the data hub every 5 s. Since oil platforms have between six to 20 of these machines, a conservative estimate provided by the partner yielded that, on average, 43,200,000 measurements are collected on a daily basis. Furthermore, as the industry partner already exploits more than 70 platforms, a conservative estimate is that every 5 s, more than 100,000 sensor measurements should be processed by the central data hub. Hence, the dataset available for processing has more than 1 × 10^12^ measurements per year. This characteristic imposes extra requirements on the low computational complexities of the algorithms and methods to be applied, as well as on the supporting computational engine.

In order to deal with such an amount of noisy data, time series segmentation is identified as a necessary technique to be used as a preprocessing step for time series analysis. This step must be able to identify blocks of homogeneous data that can be analyzed in a separate fashion. However, the massive amount of data to be processed in an on-line fashion poses a challenge to current time series segmentation methods. Consequently, we proposed a novel segmentation algorithm that was able to correctly identify those blocks of data at a viable computational cost.

Due to the lack of labeled data for the training and validation of models, we propose a solution for the detection of anomalies in turbomachinery that relies on using a one-class support vector machine (SVM) [9]. The one-class SVM learns a region that contains the training data instances (a boundary). Kernels, such as radial basis functions (RBF) [10], linear, Fourier, etc. [11], can be used to learn complex regions. For each test instance, the basic technique determines if the test instance falls within the learned region. If a test instance falls within the learned region, it is declared as normal; else it is declared as anomalous. We combine this technique with a time series segmentation to prune noisy, unreliable and inconsistent data.

Therefore, the novelty of our approach is the combination of a fast and high-quality segmentation algorithm with a one-class support vector machine approach for efficient anomaly detection.

The remainder of this paper is organized as follows. In the next section, we discuss some related work. Subsequently, we describe our proposal in detail. After that, we present a case study for offshore oil platform turbomachinery. This case study is devised to compare our approach with alternatives methods of anomalies or outlier detection. In the final section of the paper, some conclusive remarks and directions for future work are put forward.

## 2. Foundations

The present work addresses the problem of anomaly detection by combining a one-class SVM classifier that has previously been used with success for anomaly detection with a novel and fast segmentation algorithm specially devised for this problem. In this section, we present the theoretical pillars supporting the proposal.

### 2.1. Anomaly Detection

Fault and damage prevention is known as the problem of finding patterns in data that do not conform to an expected behavior [2]. Unexpected patterns are often referred to as anomalies, outliers or faults, depending on the application domain. In broad terms, anomalies are patterns in data that do not conform to a well-defined normal behavior [12]. There are also extensive surveys of anomaly detection techniques.

Anomaly detection techniques have been proposed in the literature, based on distribution, distance, density, clustering and classification. Their applications vary depending on the user, the problem domains and even the dataset. In many cases, the anomaly detection is related to outlier detection. In statistics, outliers are data instances that deviate from a given sample in which they occur. Grubbs in [13] defined them as follows: an outlying observation, or “outlier”, is one that appears to deviate markedly from other members of the sample in which it occurs.

Anomaly detection techniques can be summarized by grouping them into a sets of similar approaches:

- Distribution-based approaches: A given statistical distribution is used to model the data points [7]. Then, points that deviate from the model are flagged as anomalies or outliers. These approaches are unsuitable for moderately high-dimensional datasets and require prior knowledge of the data distribution. They are also called parametric and non-parametric statistical modeling.
- Depth-based approaches: This computes the different layers of convex hulls and flag objects in the outer layer as anomalies or outliers [14]. It avoids the requirement of fitting a distribution to the data, but has a high computational complexity.
- Clustering approaches: Many clustering algorithms can detect anomalies or outliers as elements that do not belong, or that are near, to any cluster [15,16].
- Distance-based approaches: Distance-based anomalies or outlier detection marks how distant an element is from a subset of the elements closest to it. It has been pointed out [17] that these methods cannot cope with datasets having both dense and sparse regions, an issue denominated the multi-density problem.
- Density-based approaches: Density-based anomalies or outlier detection have been proposed to overcome the multi-density problem by means of the local outlier factor (LOF). LOF measures the degree of outlierness for each dataset element and depends on the local density of its neighborhood. This approach fails to deal correctly with another important issue: the multi-granularity problem.The local correlation integral (LOCI) method, and its outlier metric, the multi-granularity deviation factor (MDEF), were proposed with the purpose of correctly dealing with multi-density and multi-granularity [18].
- Spectral decomposition: Spectral decomposition is used to embed the data in lower dimensional subspace in which the data instances can be discriminated easily Many techniques based on principal component analysis (PCA) have emerged [19]. Some of them decompose space into normal, anomaly and noise subspaces. The anomalies can be then detected in the anomaly subspace [20].
- Classification approaches: In this case, the problem is posed as the identification of which categories to which an observation belongs. It operates in two phases: first, it learns a model based on subset observations (training set), and second, it infers a class for new observations (testing set) based on the learned model. This method operates under the assumption that a classifier distinguishes between normal and anomalous classes that can be learned in the given feature space. Based on the labels available for the training phase, anomaly detection techniques based on classification can be grouped into two broad categories: multi-class [21] and one-class anomaly detection techniques [22].

### 2.2. Time Series Segmentation

In the problem of finding frequent patterns, the primary purpose of time series segmentation is dimensionality reduction. For the anomaly detection problem in turbomachines, it is necessary to segment the dataset available in order to automatically discover how the operational regime of the machine in the recent past was. There is a vast amount of work that has been done in time series segmentation, but let us state a segmentation definition and describe the available segmentation method classification, before starting to cite them.

In general terms, a time series can be expressed as a set of time-ordered possible infinite measurements [23], 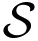, that consists of pairs 〈*s_i_*,*t_i_*〉 of sensor measurements, *s_i_*, and time instants, *t_i_*, such that,

(1) $$S=\left\{\langle s_{0},t_{0}\rangle ,\langle s_{1},t_{1}\rangle ,\ldots \langle s_{i},t_{i}\rangle ,\ldots \right\},i\in {\text{N}}^{+};\forall t_{i},t_{j}:t_{i}<t_{j}\text{if}i<j$$

Sensor measurements *s_i_* take values on a set that depends on the particular characteristics of the sensor.

In practice, time series frequently have a simpler definition as: measurements that are usually obtained at equal time intervals between them. This type of time series is known as a regular time series. In this case, the explicit reference to time can be dropped and exchanged for an order reference index, leading to a simpler expression:

(2) $$S=\left\{s_{0},s_{1}\ldots s_{i},\ldots \right\},i\in {\text{N}}^{+}$$

The use of regular time series is so pervasive that the remainder of this paper will deal only with them. Henceforth, the term, time series, will be used to refer to a regular time series.

Depending on the application, the goal of the segmentation is to locate stable periods of time, to identify change points or to simply compress the original time series into a more compact representation. Although in many real-life applications, a lot of variables must be simultaneously tracked and monitored, most of the segmentation algorithms are used for the analysis of only one time-variant variable.

A segmentation algorithm can be represented as a function Θ(·) that creates *K* segments of time series, such that,

(3) $$\Theta :S\to \langle S_{1},S_{2},\ldots ,S_{K}\rangle$$

where 〈 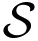
_1_, 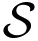
_2_, …, 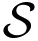
*_k_*〉exhibit the properties: (i) $S=\cup_{i=1}^{K}S_{i}$, or, in other words, that 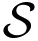 can be reconstructed from the segmentation without data loss; and (ii) 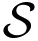
*_i_* ∩ 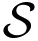
*_j_* = ∅, ∀*i*, *j* = 1,…, *K* and *i* ≠ *j*, which implies that each segment is disjoint with regard to the rest.

There is a vast literature about segmentation methods for different applications. Basically, there are mainly three categories of time series segmentation algorithms using dynamic programming. Firstly, there are sliding windows [24,25], top-down [26] and bottom-up [27] strategies.

The sliding windows method is a purely implicit segmentation technique. It consists of a segment that is grown until it exceeds some error bound. This process is repeated with the next data point not included in the newly approximated segment. However, like all implicit methods, it is extremely slow and not useful for real-time applications; its complexity is *O*(*Ln***_
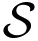
_**), where *n***_
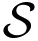
_** is the number of elements of 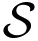 (*n***_
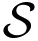
_** =| 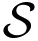
|) and *L* = *n***_
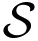
_**/*K* is the average segment length.

Top-down methods are those where the time series is recursively partitioned until some stopping criteria is met. This method is faster than the sliding window method above, but it is still slow; the complexity is *O*(*n*^2^*K*). Additionally, the bottom-up starts from the finest possible approximation, and segments are merged until some stopping criteria is met.

Later, during the process of approximating a time series with straight lines, there are at least two ways of finding the approximating line: linear interpolation and linear regression [28]. Linear interpolation tends to closely align the endpoint of consecutive segments, giving the piecewise approximation a “smooth” look. In contrast, piecewise linear regression can produce a very disjointed look on some datasets. However, the quality of the approximating line, in terms of Euclidean distance, is generally better in the regression approach [29].

There are also more novel methods, for instance those using clustering for segmentation. The clustered segmentation problem is clearly related to the time series clustering problem [30], and there are also several definitions for time series [31,32]. One natural view of segmentation is the attempt to determine which components of a dataset naturally “belong together”.

There exist two classes of algorithms for solving the clustered segmentation problem. The distance-based clustering of segmentations measures the distance between sequence segmentations. In our approach, we employ a standard clustering algorithm (e.g., k-means) on the pair-wise distance matrix. The second class of algorithms consists of two randomized algorithms that cluster sequences using segmentations as “centroids”. In particular, we use the notion of a distance between a segmentation and a sequence, which is the error induced on the sequence when the segmentation is applied to it. The algorithms of the second class treat the clustered-segmentation problem as a model selection problem and then try to find the best model that describes the data.

There also methods considering multiple regression models. In [33], a segmented regression model is considered with one independent variable under the continuity constraints, and the asymptotic distributions of the estimated regression coefficients and change points are studied. In [34-36], some special cases of the model studied cited before are considered, and more details on the distributional properties of the estimators are provided. Bai [37–39] considered a multiple regression model with structural changes, a model without the continuity constraints at the change points, and studied the asymptotic properties of the estimators.

## 3. Algorithm Proposal

As already hinted earlier, our proposal combines a fast segmentation algorithm with a support vector machine one-class classifier. The segmentation algorithm takes care of identifying relatively homogeneous parts of the time series in order to focus the attention of the classifier on the most relevant portion of the time series. Therefore, parts of the time series that remain in the past can be safely disregarded.

### 3.1. Problem Formalization

**Figure 1.**
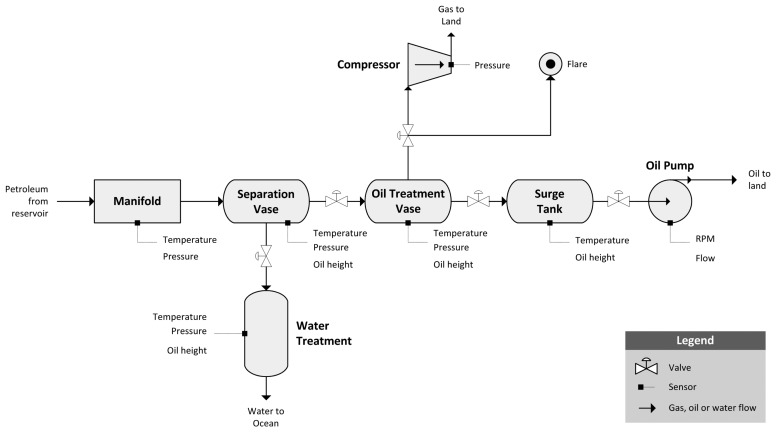
Oil platform process plant work-flow.

Nowadays, it is common that offshore oil platforms use equipment control automation to act upon perceived events. This equipment control automation includes sensors for monitoring equipment behavior and remotely-controlled valves. Plant automation physically protects plant integrity and acts by reacting to anomalous conditions. Equipment usage is automatically controlled by a priori limit values, usually provided by the equipment manufacturer, that establish an operational interval. Figure 1 presents a general operational workflow of an oil platform, detailing the main components and processes.

Assuming independence between turbomachines and that their sensors operate in a reliable and consistent mode, we can deal with each one separately. Although, in practice, different machines do affect each other, as they are interconnected, for the sake of simplicity, we will be dealing with one at a time.

Using this scheme, we can construct an abstract model of the problem. A given turbomachine, 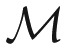, is monitored by a set of *m* sensors *s*^(^*^j^*^)^ ∈ 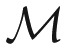, with *j* = 1, …, *m*. Each of these sensors are sampled at regular time intervals in order to produce the time series:

(4) $$S_{t_{\max},t_{0}}^{\left(j\right)}:=\left\{s_{t}^{\left(j\right)}\right\},t_{0}\leq t\leq t_{\max}$$

Using this representation and assuming that sensors are independent, the problem of interest can be expressed as a two-part problem: (i) to predict a future anomaly in a sensor; and (ii) to perform an action based on anomaly predictions (decision-making). This can be expressed more formally as:

#### Definition 1 (Sensor Anomaly Prediction)

*Find a set of anomaly prediction functions, A*^(^*^j^*^)^(·), *Such That:*

(5) $$A^{\left(j\right)}\left(S_{t,t-\Delta t}^{\left(j\right)}|{\hat{S}}_{t_{\max},t_{0}}^{\left(j\right)}\right)=\{\begin{array}{l} 1\text{predictedanomaly}\\ 0\text{in other case} \end{array}$$

*that is constructed using a given reference (training) set of sensor data*, ${\hat{S}}_{t_{\max},t_{0}}^{\left(j\right)}$, *and determines if there will be a failure in the near future by processing a sample of current sensor data* $S_{t,t-\Delta t}^{\left(j\right)}$, *with t_max_* < *t* – Δt < *t and, generally*, Δ*t* ≫ *t_max_* – *t*_0_. *Using those functions, the second problem can be stated as:*

#### Definition 2 (Machine Anomaly Alarm)

*For each turbomachine* ***
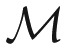
***, *obtain a machine alarm function:*

(6) $$F_{M}\left(a_{t}^{\left(1\right)},\ldots a_{t}^{\left(m\right)}|w_{M}\right)=\{\begin{array}{l} 1\text{alarm signal}\\ 0\text{in other case} \end{array}$$

*where* $a_{t}^{\left(j\right)}=A^{\left(j\right)}\left(S_{t,t-\Delta t}^{\left(j\right)}\right)$ *and the weights vector, w***_
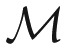
_** = {*w*^(1)^,…,*w*^(^*^m^*^)^}, *represents the contribution, or relevance, of each sensor to an alarm firing decision*.

It must be noted that, although we have expressed these problems in a crisp (Boolean) form, they can be expressed in a continuous [0, 1] form suitable for the application of fuzzy logic or other forms of uncertainty reasoning methods. The discussion of those approaches and their application is out of the scope of this paper.

In order to synthesize adequate *A*^(^*^j^*^)^ and *f***_
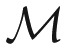
_**, it is necessary to identify the different operational modes of the the machine. Knowing the operational modes of the machine enables the creation of *A*^(^*^j^*^)^ and *f***_
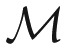
_** functions, either explicitly or by means of a modeling or machine learning method, that correctly respond to each mode.

### 3.2. Segmentation Algorithm

In this section, we introduce a novel and fast algorithm for time series segmentation. Besides the obvious purpose of obtaining a segmentation method that produces low approximation errors, another set of guidelines were observed while devising the algorithm. They can be summarized as follows:

- Low computational cost: The application context calls for algorithms capable of handling large amounts of data and that scale properly as the those amounts are increased. Most current segmentation algorithms have such a computational complexity, that it impairs them from correctly tackling the problems of interest.
- Easy parametrization: One important drawback of current approaches is that their parameters may be hard to set by the end users. In our case, we have as the main parameter the significance test threshold, which is very easy to understand feature.

**Figure 2.**
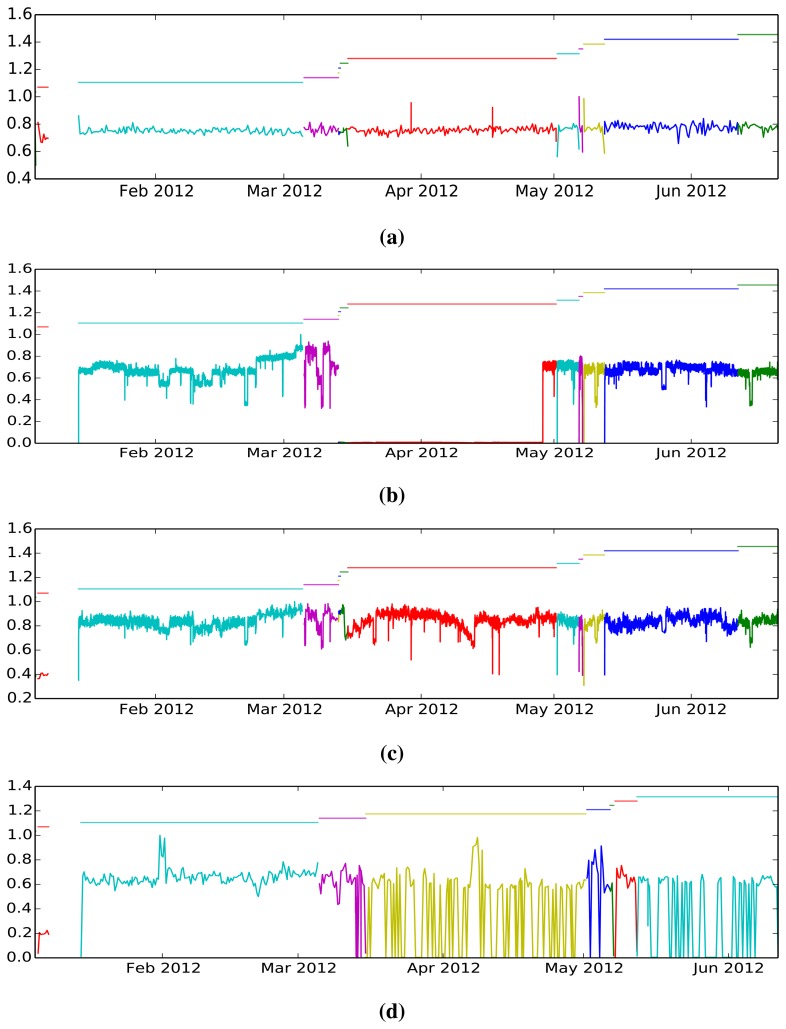
Pseudocode of yet another segmentation algorithm (YASA).

Relying on those principles, we propose yet another segmentation algorithm (YASA). YASA is presented in Figure 2 in schematic form. The algorithm is best understood when presented in recursive form. A call to the segmentation procedure first checks if the current level of recursion is acceptable. After that, it fits a linear regression to the time series data. If the regression passes the linearity statistical hypothesis test, then the current time series is returned as a unique segment.

If the regression does not model the data correctly, this means that it is necessary to partition the time series into at least two parts that should be further segmented. The last part of YASA is dedicated to this task. It locates the time instant, t_s_, where the regression had the largest error residuals. It also guarantees that that time instant does not create an excessively short time series chunk. Once an adequate time instant is located, it is used as a split point to carry out the segmentation of the parts of the time series located on both sides of it.

### 3.3. One-Class Support Vector Machine

The problem, as it is posed, implies determining whether (new) sensor data belong to a specific class, determined by the training data, or not.

To cope with this problem, one-class classification problems (and solutions) are introduced. By just providing the normal training data, an algorithm creates a (representational) model of this data. If newly encountered data is too different, according to some measurement from this model, it is labeled as out-of-class.

Support vector machines (SVMs) can be used for the problem described above. SVMs are supervised learning models with associated learning algorithms that analyze data and recognize patterns. SVMs have been successfully used for classification and regression analysis. Given a set of training examples, each marked as belonging to one of many categories, an SVM training algorithm builds a model that assigns new examples into one category or the other, making it a non-probabilistic binary linear classifier. An SVM model is a representation of the examples as points in space, mapped so that the examples of the separate categories are divided by a clear gap that is as wide as possible. More precisely, a support vector machine constructs a hyperplane or set of hyperplanes in a high- or infinite-dimensional space, which can be used for classification, regression or other tasks. Intuitively, a good separation is achieved by the hyperplane that has the largest distance to the nearest training data point of any class (the so-called functional margin), since, in general, the larger the margin, the lower the generalization error of the classifier.

In order to understand one-class SVMs, it is convenient to first examine the traditional two-class support vector machine. Consider a (possibly infinite) dataset,

(7) $$\Psi =\left\{\langle {\text{x}}_{1},y_{1}\rangle ,\langle {\text{x}}_{2},y_{2}\rangle ,\ldots \langle {\text{x}}_{i},y_{i}\rangle ,\ldots \right\}$$

where ***x****_i_* ∈ ℝ*^n^* is a given data point and *y_i_* ∈ {1, 1} is the *i*-th output pattern, indicating the class membership.

SVMs can create a non-linear decision boundary by projecting the data through a non-linear function *φ*() to a space with a higher dimension. This implies that data points, which cannot be separated by a linear threshold in their original (input) space, are converted to a feature space 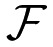, where there is a hyperplane that separates the data points of one class from another. When that hyperplane is projected back to the original space, it has the shape of a non-linear curve. This hyperplane is represented by the equation,

(8) $$\begin{array}{ccc} {\text{w}}^{T}\text{x}+\text{b}=0, & \text{with w}\in F, & \text{b}\in {\text{R}}^{n} \end{array}$$

The hyperplane that is constructed determines the border between classes. All of the data points for the class “− 1” are on one side, and all of the data points for class “1” are on the other. The distance from the closest point from each class to the hyperplane is equal; thus, the constructed hyperplane searches for the maximal margin between the classes.

Slack variables, *ξ_i_*, are introduced to allow some data points to lie within the margin in order to prevent the SVM classifier from over-fitting the noisy data (or creating a soft margin). Constant *C* > 0 determines the trade-off between maximizing the margin and the number of training data points within that margin (and, thus, training errors). Posed as an optimization problem, the adjustment of an SVM has as the objective to minimize the problem,

(9) $$\begin{array}{l} \begin{array}{cc} \text{minimize} & f(\text{w},\text{b},\text{xi})=\frac{{\left\|\text{w}\right\|}^{2}}{2}+C\sum_{i=1}^{n}\xi_{i}, \end{array}\\ \begin{array}{ccc} \text{subject to} & y_{i}(w^{T}\phi (x_{i})+b)\geq 1-\xi_{i}, & \forall i=1,\ldots ,n; \end{array}\\ \begin{array}{cc} & \xi_{i}\geq 0. \end{array} \end{array}$$

Solving Equation (9) using quadratic programming, the decision function (classification) rule, *c*(***x***), for a data point ***x***, becomes:

(10) $$c(\text{x})=\text{sign}\left(\sum_{i=1}^{n}\alpha_{i}y_{i}K(\text{x},{\text{x}}_{i})+b\right)$$

Here, the *α_i_* > 0 are the Lagrange multipliers that weight the decision function and, thus, the “support” machine; hence, the name support vector machine. Since SVMs are generally considered to be sparse, there will be relatively few Lagrange multipliers with a non-zero value. Function *K*(***x***, ***x****_i_*) is known as the kernel function. Popular choices for the kernel function are linear, polynomial and sigmoidal. However, the most popular choice by far, provided that there is not enough *a priori* knowledge about the problem, is the Gaussian radial basis function:

(11) $$K(\text{x},{\text{x}}^{\prime })=\exp\left(-\frac{{\left\|x-x^{\prime }\right\|}^{2}}{2\sigma^{2}}\right)$$

where *σ* ∈ ℝ, is the kernel parameter and ‖·‖ is the dissimilarity measure. This is derived from the fact that this kernel function is able to model a non-linear decision boundary with relatively simple mathematical tools. Furthermore, Gaussian kernels are universal kernels. This means that their use with appropriate regularization guarantees a globally optimal predictor, which minimizes both the estimation and approximation errors of a classifier.

One-class classification-based anomaly detection techniques assume that all training instances have only the same class label. Then, a machine learning algorithm is used to construct a discriminative boundary around the normal instances using a one-class classification algorithm. Any test instance that does not fall within the learned boundary is declared as an anomaly. Support vector machines (SVMs) have been applied to anomaly detection in the one-class setting. One-class SVMs find a hyperplane in feature space, which has the maximal margin to the origin, and a preset fraction of the training examples lays beyond it.

The support vector method for novelty detection [40] essentially separates all of the data points from the origin (in feature space 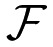) and maximizes the distance from this hyperplane to the origin. This results in a binary function that captures regions in the input space where the probability density of the data lives. Thus, the function returns “+1” in a reduced region (capturing the training data points) and “−1” elsewhere.

The quadratic programming minimization problem is slightly different from that previously stated, but the similarity is evident,

(12) $$\begin{array}{ll} \text{minimize} & f(\text{w},{\text{x}}_{i},\rho )=\frac{1}{2}{\left\|\text{w}\right\|}^{2}+\frac{1}{\nu n}\sum_{u=1}^{n}\xi_{i}-\rho ,\\ \text{subject to} & \text{w}\cdot \phi ({\text{x}}_{i})\geq \rho -\xi_{i},\\ & \xi_{i}\geq 0,\\ & \forall i-1,\ldots ,n. \end{array}$$

Applying the Lagrange techniques and using a kernel function for the dot-product calculations, the decision function becomes:

(13) $$c(\text{x})=\text{sign}((\text{w}\cdot \phi ({\text{x}}_{i}))-\rho )=\text{sign}\left(\sum_{i=1}^{n}K(\text{x},{\text{x}}_{i})-\rho \right)$$

This method thus creates a classification hyperplane characterized by w and *ρ*, which has maximal distance from the origin in feature space 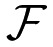 and separates all of the data points from the origin. Another method is to create a circumscribing hypersphere around the data in feature space.

In this paper, we have applied this approach combined with an evolutionary algorithm [41] for optimizing the maximal margin, as well as other SVM parameters, with respect to outlier detection accuracy.

## 4. Anomaly Detection in Offshore Oil Extraction Turbomachines

In order to validate our approach, it was necessary to perform two comparative and validation experiments: one that focused on the segmentation algorithm and its performance compared with other state-of-the-art alternatives and other that had to do with the anomaly detection method as a whole. This section of the paper describes these experiments and their outcome.

### 4.1. Comparative Experiments for Time Series Segmentation

YASA is currently being applied with success to the problem of segmenting turbomachine sensor data of a major petroleum extraction and processing conglomerate of Brazil. In this section, we present a part of the experimental comparison involving some of the current state-of-the-art methods and our proposal, which was carried out in order to validate the suitability of our approach. Readers must be warned that the results presented here had to be transformed in order to preserve the sensitive details of the data.

For these experiments, we selected a dataset from sensors of the measurements taken with a five-minute frequency obtained during the first half of the year 2012 (from 1 January 2012, 00:00, to 30 June 2012, 23:59) from more than 250 sensors connected to an operational turbomachine. An initial analysis of the data yields that there are different profiles or patterns that are shared by different sensors. This is somewhat expected, because sensors with similar purposes or supervising similar physical properties should have similar reading characteristics.

**Figure 3.**
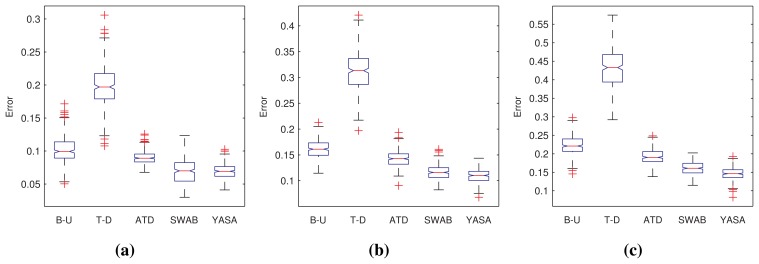
A sample of the four main types of time series contained in the dataset. We have marked with color changes the moments in which the machine was switched on/off. (**a**) Homogeneous time series; (**b**) unstable/noisy time series; (**c**) multi-modal series; (**d**) highly unstable time series, probably caused by faulty sensors.

Figure 3 displays the four prototypical example time series profiles found in the dataset. First, in Figure 3a, we have smooth and homogeneous time series that are generally associated with slow-changing and stable physical properties. Second, in Figure 3b, we found fast-changing, unstable sensor readings that could be a result of sensor noise, sensor malfunction or unstable physical quantity. There is a third class of time series, such as the one presented in Figure 3c, which exhibits a clear change in operating profile, attributable either to different operational regimes of the machine or the overall extraction/processing process. Finally, there is a class of sensors that are extremely unstable that contradict the *a priori* working principles of the machine itself. It must be noted that, in each case, we have marked with a color transition the moment in which the machine transitioned from “on” to “off” states and *vice versa*.

Using this dataset, we carried out a study comparing four of the main segmentation algorithms and our proposal. In particular, we compare bottom-up [27], top-down [42], adaptive top-down [26] and sliding window and bottom-up algorithms [29].

The need for comparing the performance of the algorithms when confronted with the different sensor data prompts the use of statistical tools. These tools are used in order to reach a valid judgment regarding the quality of the solutions; to compare different algorithms with each other and their computational resource requirements.

Box plots [43] are one such representation and have been repeatedly applied in our context. However, box plots allow a visual comparison of the results, and in principle, some conclusions could be deduced out of them.

**Figure 4.**
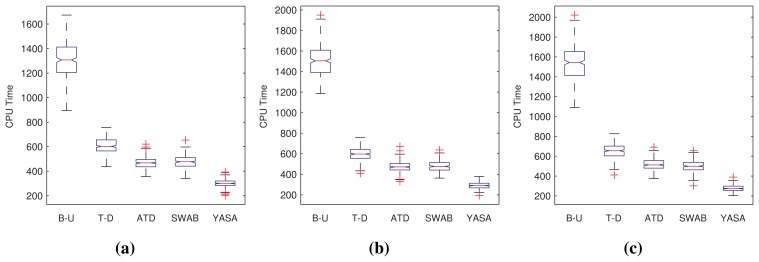
Box plots of the root mean squared errors yielded by the bottom-up (B-U), top-down (T-D), adaptive top-down (ATD), sliding window and bottom-up (SWAB) and our proposal (YASA). Data have been transformed for sensitivity reasons. (**a**) Errors for homogeneous series; (**b**) errors for multi-modal series; (**c**) errors for noisy series.

Figure 4 shows the quality of the results in terms of the mean squared error obtained from the segmentation produced by each algorithm in the form of box plots. We have grouped the results according to the class of sensor data for the sake of a more valuable presentation of the results. The main conclusion to be extracted from this initial set of results is that our proposal was able to achieve a similar performance, and, in some cases, a better performance, when compared with the other methods.

The statistical validity of the judgment of the results calls for the application of statistical hypothesis tests. It has been previously remarked by different authors that the Mann-Whitney-Wilcoxon U-test [44] is particularly suited for experiments of this class. This test is commonly used as a non-parametric method for testing the equality of population medians. In our case, we performed pair-wise tests on the significance of the difference of the indicator values yielded by the execution of the algorithms. A significance level, α, of 0.05 was used for all tests.

Table 1 contains the results of the statistical analysis, which confirm the judgments put forward before.

**Figure 5.**
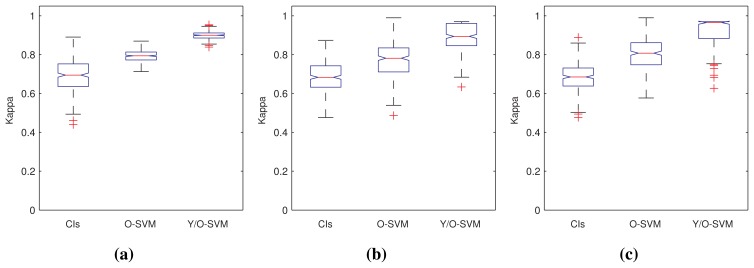
Box plots of the CPU time needed by the B-U, T-D, ATD, SWAB and our proposal (YASA). Data have been transformed for sensitivity reasons. (**a**) Errors for homogeneous series; (**b**) errors for multi-modal series; (**c**) errors for noisy series.

Comparing performance is clearly not enough, as one of the leitmotifs of this work is to provide a good and fast segmentation algorithm. That is why we carry out a similar study as the previous one, this time focusing on the amount of CPU time required by each algorithm. Figure 5 summarizes this analysis. It is visible how our approach required less computation to carry out the task. Table 2 allows one to assert this analysis with the help of statistical hypothesis tests, as explained in the previous analysis.

### 4.2. Comparative Experiments for Anomaly Prediction

In order to experimentally study and validate our approach, we carried out a study involving a real-world test case. In this case, we dealt with a dataset of measurements taken with a five-minute frequency obtained during the first half of the year 2012 from 64 sensors connected to an operational turbomachine. An initial analysis of the data yields that there are different profiles or patterns that are shared by different sensors. This is somewhat expected, as sensors with similar purposes or supervising similar physical properties should have similar reading characteristics.

There are at least three time series profiles in the dataset. On the one hand, we have smooth homogeneous time series that are generally associated with slow-changing physical properties. Secondly, we found fast changing/unstable sensor readings that could be a result of sensor noise or unstable physical quantity. There is a third class of time series, which exhibit a clear change in operating profile attributable to different operational regimes of the machine or the overall extraction/processing process.

In order to provide a valid ground for comparison, we tested the method currently used by the platform operator, which is based on statistical confidence intervals [45], a one-class support vector machine-based classifier, as described earlier in this work, and our proposal. Problem data were transformed to detect an anomaly based on consecutive sensor measurements in one hour.

The approach in current use was not (and cannot be) fully disclosed, as it is business sensitive information. However, in broad terms, for each sensor, this method receives a sample data chunk, which has been selected by an expert as a valid one. It filters out outlier elements and computes the confidence intervals at a predefined percent of the resulting dataset. A possible failure is detected when a given set of sensor measurements are consistently outside such an interval.

We carried out this task by creating an experimental dataset, which contains 20 anomaly instances extracted from each of the 64 time series and 20 regular or non-anomalous situations.

**Figure 6.**
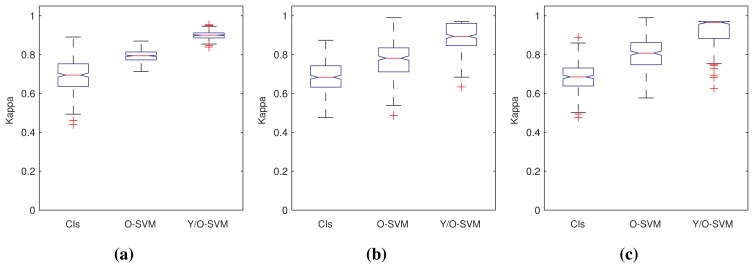
Box plots of the Kappa statistic yielded by each class of dataset. (**a**) Errors for homogeneous series; (**b**) errors for multi-modal series; (**c**) errors for noisy series.

Figure 6 shows the quality of the results in terms of the Kappa statistic [46] obtained from each algorithm in the form of box plots. We have grouped the results according to the class of sensor data for the sake of a more valuable presentation of results.

The statistical validity of the judgment of the results calls for the application of statistical hypothesis tests [47]. The McNemar test [48] is particularly suited for the assessment of classification problem results, like the ones addressed here. This test is a normal approximation used on paired nominal data. It is applied to 2 × 2 contingency tables with a dichotomous trait, with matched pairs of subjects, to determine whether the row and column marginal frequencies are equal. In our case, we applied the test to the confusion matrices performing pair-wise tests on the significance of the difference of the indicator values yielded by the executions of the algorithms. A significance level, α, of 0.05 was used for all tests.

Table 3 contains the results of the statistical analysis, which confirm the judgments put forward before. In particular, it is notable that the combination of YASA and a one-class SVM was able to outperform the other two approaches in all problem instances, with the exception of the homogeneous series. In this case, it produced similar results to the “plain” SVM and outperformed the confidence interval approach. This makes a certain sense, as in homogeneous data, there is relatively little need for segmentation, and it can be hypothesized that our approach tends to perform similarly to just using an SVM. It is also interesting that, in the homogeneous time series dataset, confidence intervals yielded better results than SVM. This can be a fact also derived from the nature of the data, which can be better captured through this technique.

In any case, in the more complicated problems, the ability of the methodology put forward earlier in this work to detect those anomalies is clear.

## 5. Conclusions

This work describes a comprehensive approach to anomaly detection in the context of the oil industry. Specifically, we have dealt with the problem of detecting anomalies in turbomachines used in offshore oil platforms relying on sensor data streams (time series). This problems posed some challenges derived from the amount of data that must be processed.

In order to cope with these problem characteristics, we proposed a novel segmentation algorithms, which we called YASA, and coupled it with a one-class support vector machine. YASA is a fast segmentation algorithm that has the additional feature of being easily parametrized. YASA takes care of identifying homogeneous sections of the sensor time series. Those sections are then fed to a one-class SVM. This one-class SVN creates a model of valid sensor signals. Consequently, this model is used to detect sensor measurements that do not conform with it and, hence, represent anomalous situations.

The methods being put forward have been assessed with some of the alternatives in a real-case scenario with the purpose of studying its validity, viability and performance. In particular, we have compared our method with the approach currently used by our industry partner, as well as the straightforward use of one-class SVMs. These methods were applied to a reduced problem that consisted of the supervision of a single turbomachine. The outcome of this experiment shows that the combination of YASA and one-class SVM was able to outperform the other approaches. Similarly, it is notable that YASA exhibited a smaller computational footprint than its alternative segmentation algorithms.

It is important to underline that an important feature of YASA has to do with simple parametrization and usability. Most other methods require having an *a priori* number of segments (or number or recursion levels) as the input. This implies a severe drawback, as knowing the amount of segments in a time series is almost the same problem as segmenting it. Therefore, an incorrect setting of those parameters would certainly have a negative bias on the outcome of the method. On the other hand, YASA uses a statistical hypothesis test as the criterion. This has the important consequence that setting the algorithm parameters becomes quite simple and intuitive. Furthermore, this scheme should add or reduce the number of segments accordingly.

An automatic supervision system, whose essential element is the method described in this paper, is currently deployed by a major petroleum industry conglomerate of Brazil. In this sense, our approach was able to outperform the current approach used in the production system, as well as the traditional formulation of a one-class support vector machine (SVM).

Further work on this topic is called for and is currently being carried out. An important direction is the formal understanding of the computational complexity of the proposal and, particularly, of YASA. The use of a statistical hypothesis test as the stop criterion for the algorithm complicated the straightforward deduction of the complexity. It is also important to propose methods for combining anomaly evidence from different sensors at onetime. We are currently working on detecting situations when, in a given period of time, different sensors report slightly anomalous measurements. In this cases, separate sensor deviations are not important enough to represent an anomaly, but all of them combined could be used to deduce a near-future anomaly.

We also intend to extend the context of application to other Big Data and/or related application contexts. More specifically, we are applying YASA and one-class SVMs to the problem of automatic activity recognition based on smart phone sensors. We also intend to extrapolate these results to data fusion applications for aerial and maritime vehicle tracking.

## Figures and Tables

**Table 1.** Results of the statistical hypothesis tests on segmentation errors. Cells marked in green ( 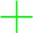) are cases where a statistically-significant difference was observed. Red cells ( 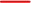) denote cases where the results of both algorithms were statistically homogeneous.

	**Top-Down**	**Bottom-Up**	**Adaptive T-D**	**SWAB**	**YASA**
**Homogeneous series**					

Top-Down	·	+	−	−	−
Bottom-Up		·	+	+	+
Adaptive Top-Down			·	+	−
Sliding Window and Bottom-up				·	−
YASA					·
**Multi-modal series**					

Top-Down	·	+	−	−	−
Bottom-Up		·	+	+	+
Adaptive Top-Down			·	+	−
Sliding Window and Bottom-up				·	−
YASA					·
**Noisy series**					

Top-Down	·	−	+	−	−
Bottom-Up		·	+	+	+
Adaptive Top-Down			·	+	−
Sliding Window and Bottom-up				·	−
YASA					·
**All data**					

Top-Down	·	−	+	−	−
Bottom-Up		·	+	+	+
Adaptive Top-Down			·	+	−
Sliding Window and Bottom-up				·	−
YASA					·

**Table 2.** Results of the statistical hypothesis tests on the CPU time required to perform the segmentation. Green cells ( 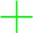) mark cases where the results of both algorithms were statistically different. Cells marked in red ( 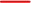) are cases where no statistically-significant difference was observed.

	**Top-Down**	**Bottom-Up**	**Adaptive T-D**	**SWAB**	**YASA**
**Homogeneous series**					

Top-Down	·	+	+	+	+
Bottom-Up		·	+	+	+
Adaptive Top-Down			·	−	+
Sliding Window and Bottom-up				·	+
YASA					·
**Multi-modal series**					

Top-Down	·	+	+	+	+
Bottom-Up		·	+	+	+
Adaptive Top-Down			·	−	+
Sliding Window and Bottom-up				·	+
YASA					·
**Noisy series**					

Top-Down	·	+	+	+	+
Bottom-Up		·	+	+	+
Adaptive Top-Down			·	−	+
Sliding Window and Bottom-up				·	−
YASA					·
**All data**					

Top-Down	·	+	+	+	+
Bottom-Up		·	+	+	+
Adaptive Top-Down			·	−	+
Sliding Window and Bottom-up				·	+
YASA					·

**Table 3.** Results of the McNemar statistical hypothesis tests. Green cells ( 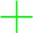) denote cases where the algorithm in the row statistically was better than the one in the row. Cells marked in red ( 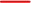) are cases where the method in the column yielded statistically better results when compared to the method in the row. Finally, cells in blue ( 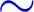) denote cases where results from both methods were statistically indistinguishable.

	**Y+OSVM**	**OSVM**	**CIs**
**Homogeneous series**			

YASA + One-class SVM (Y + OSVM)	·	∼	+
One-class SVM (OSVM)		·	−
Confidence intervals (CIs)			·
**Multi-modal series**			

YASA + One-class SVM (Y + OSVM)	·	+	+
One-class SVM (OSVM)		·	∼
Confidence intervals (CIs)			·
**Noisy series**			

YASA + One-class SVM (Y + OSVM)	·	+	+
One-class SVM (OSVM)		·	∼
Confidence intervals (CIs)			·
**All data**			

YASA + One-class SVM (Y + OSVM)	·	+	+
One-class SVM (O-SVM)		·	+
Confidence intervals (CIs)			·
